# Perspectives on public involvement in health research from Singapore: The potential of a supported group model of involvement

**DOI:** 10.1111/hex.13058

**Published:** 2020-06-10

**Authors:** Lidia Luna Puerta, Bernadette Bartlam, Hsiao‐Li Shirley Sun, Helen E. Smith

**Affiliations:** ^1^ Family Medicine and Primary Care Lee Kong Chian School of Medicine Nanyang Technological University Singapore Singapore Singapore; ^2^ School of Social Sciences Nanyang Technological University Singapore Singapore Singapore; ^3^ Division of Public Health and Primary Care Brighton and Sussex Medical School Brighton UK

**Keywords:** Asia, attitudes, cultural contexts, health research, patients, PPI, public, public involvement, qualitative methods, qualitative research, research design, Singapore

## Abstract

**Background:**

Singapore is an international research hub, with an emphasis on translational clinical research. Despite growing evidence of the positive impact of public involvement (PPI) in research, it remains rare in Singapore.

**Aims:**

1. To investigate Singaporean public perspectives around the rationale, role and scope for being involved in health research
2. To identify the potential, challenges, facilitators and strategies for implementing PPI in Singapore.

**Design:**

Semi‐structured qualitative interviews with members of the public, analysed using thematic framework analysis.

**Results:**

Twenty people participated. Four main themes emerged: potential benefits; challenges; facilitators; and strategies for implementation. Whilst initially unfamiliar with the concept, all interviewees recognized potential benefits for the research itself and those involved, including researchers. PPI was seen to offer opportunities for public empowerment and strengthening of relationships and understanding between the public, academics and health professionals, resulting in more impactful research. Challenges included a Singaporean culture of passive citizenship and an education system that inculcates deferential attitudes. Facilitators comprised demographic and cultural changes, including trends towards greater individual openness and community engagement. Implementation strategies included formal government policies promoting involvement and informal community‐based collaborative approaches.

**Conclusion:**

Given the socio‐political framework in Singapore, a community‐based approach has potential to address challenges to PPI and maximize impact. Careful consideration needs to be given to issues of resource and support to enable members of the public to engage in culturally sensitive and meaningful ways that will deliver research best placed to effectively address patient needs.

## 1 INTRODUCTION

### 1.1 Patient and public involvement

In health research, public involvement (PPI) is defined as ‘research being carried out ‘with’ or ‘by’ members of the public rather than ‘to’, ‘about’ or ‘for’ them’.^1^ Promoting PPI in health research in Western countries began over two decades ago.^2^, ^3^ In the UK, PPI is now a required consideration for publicly funded health research studies, whilst in North America, Australia and some European countries such as Denmark and Spain, public engagement is strongly encouraged by research commissioners and national organizations.^3^, ^4^, ^5^, ^6^, ^7^, ^8^, ^9^, ^10^ An international network advocating for ‘a world where patient and public involvement is an integral part of health research’ was launched in late 2017 and has been joined by 240 organizations and individuals worldwide.^11^

PPI can inform all aspects of research, from identifying and prioritizing questions through to design, data collection, analysis and dissemination. Patients have lived experiences of conditions and their treatments, and can comment on areas of uncertainty where research is needed. Patient input can thus help in asking patient‐focused questions and engaging other patients.^12^, ^13^, ^14^ There are ethical arguments underpinning PPI which suggest it can rebalance power, giving a voice to those whose knowledge is less heard in traditional academic and clinical settings, and providing new perspectives from the public as ‘marginal knowers’ and yet ‘agents of knowledge’,^15^ ^(p233)^ who can share their expertise. A utilitarian argument has also been put forward^16^, ^17^, ^18^, ^19^ based on the premise that embedding the views and needs of patients in research is more likely to produce outcomes and interventions that will be implemented in policy and practice.^20^ Involvement can also positively impact on both patients and researchers, increasing self‐confidence, knowledge and skills.^2^, ^21^, ^22^, ^23^ Despite these arguments for PPI, barriers exist to its implementation, such as potential conflicting agendas; challenges in power differentials; lack of time and resources; varying degrees of public awareness; researchers’ attitudes and skills; lack of clear purpose, monitoring and evaluation; and wider research systems not supporting researchers’ individual efforts to involve.^4^, ^24^, ^25^, ^26^ On the other hand, the literature presents numerous facilitators, such as having clear objectives; establishing ground rules, training and support for all stakeholders; making PPI accessible to a diverse range of stakeholders; building trust; and highlighting the added value to and from those involved.^25^, ^26^, ^27^, ^28^

### 1.2 The Singapore context

Founded in 1965, Singapore has a culture that privileges the collective rather than the individual experience.^29^ The country has experienced immense economic and social change, rising from ‘Third World’ poverty^30^ to being an ‘Asian Tiger’.^31^ It has a population that has doubled in 50 years and which is multi‐ethnic, with four official languages,^32^ with English promoted as the ‘working language’.^33^ Singapore prides itself on leading global rankings in education,^34^ with a system structured to ‘foster a sense of national identity’ and *Shared Values* ^29^, ^35^, ^36^, ^37^ so as to promote unity within its diverse population. Singapore workers are amongst the hardest working in the world in terms of number of hours worked,^38^ with implications in terms of leisure time and availability for volunteering.

Singapore is an international research hub,^39^, ^40^ but PPI in health research remains rare, with the public viewed largely as ‘subjects’ providing necessary data.^41^ Singapore attracts globally renowned scholars and researchers,^39^, ^40^ and thus provides a useful case study for exploring the potential for PPI within an Asian, yet internationally diverse, context. This aims of this research were to investigate public perspectives on the rationale, role and scope for being involved in health research and to identify the potential challenges, facilitators and strategies for implementing PPI in Singapore.

## 2 METHODS

### 2.1 Research design

#### 2.1.1 Recruitment

Participants were identified in two ways: through (a) local community groups and (b) participants in a previous study,^41^ who were asked to invite potential participants to contact the researcher via telephone. Four community groups (Council for Third Age, Alzheimer's Disease Association, Parkinson Society Singapore and Muscular Dystrophy Association Singapore) were selected due to their high level of activity and wide membership; they were contacted via email and telephone to organize a talk for its members and invite them to take part in this study, but none were interested in collaborating with this research. Purposive (maximum variation) sampling was used to ensure a range of perspectives, ages and ethnic groups.^42^, ^43^, ^44^ Members of the public over 21 who were not English speakers or who had a professional role in health care were excluded. Once face‐to‐face or telephone contact was made, participants were given information on the study and, if still agreeable, arrangements were made for an interview at a time and place convenient to them. As a token of appreciation, they received a SGD15 gift voucher.

Out of 45 members of the public contacted by LLP, all of them replied and 20 agreed to be interviewed. Saturation was reached after 15 interviews, but we continued to 20 to ensure a solid representation of all age and demographic groups.

#### 2.1.2 Data collection

Data were collected through face‐to‐face semi‐structured interviews, designed to guide participants in reflecting on their experiences and views, and to enable them to produce their own narratives, privileging those issues most salient to them.^42^, ^45^, ^46^ Interviews were conducted by LLP in July and August 2018.

The topic guide was informed by published literature on the scope and practice of PPI and was adapted iteratively to include emerging themes from the analysis. The initial version included participants’ personal background, their views on research and its relevance and impact, their own experiences of research and their understanding of PPI, including how it might be operationalized locally. These broad topic areas were expanded in subsequent topic guides to explore emerging themes, particularly the opportunities and challenges of PPI involvement within the context of Singaporean identity and education, how PPI could be operationalized at each of the different stages in the research cycle and supporting researchers wishing to include PPI in their research.

Informed consent was obtained before the interview and confirmed again on completion. All interviews were audio‐recorded, transcribed verbatim, cleaned and anonymized before analysis. Data were stored securely in line with Nanyang Technological University's (NTU) requirements and the Personal Data Protection Act (2012).^47^, ^48^

#### 2.1.3 Analysis

**Figure 1:**
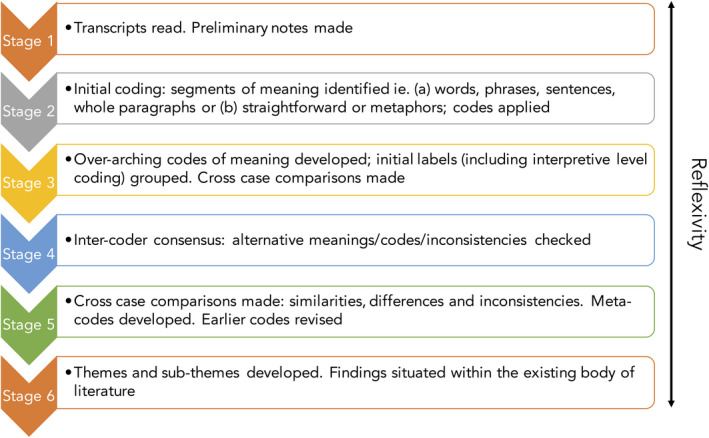
The six stages of coding

A structured thematic analysis^49^, ^50^ of the transcripts was undertaken in six stages (see Figure 1), supported by NVivo 10 data management and analysis software.^51^

Analysis started with familiarization with the data (Stage 1). An exploratory framework was adopted using applied thematic analysis, with coding occurring in distinct stages beginning with each case before moving to a cross‐case comparison and analysis (Stages 2‐3).^50^, ^52^ Analysis thus progressed from initial descriptive codes (sub‐themes) to interpretative theoretical constructs (main themes).^53^

To ensure reliability, a process of inter‐coder consensus was adopted (Stage 4), in which two of the authors (LLP and HES) independently coded a random selection of six transcripts before developing a coding framework which was applied to the remaining data by LLP. This codebook is a structured compendium of codes, providing the name of the code or theme, examples of source data and researchers' interpretive summary, providing an overview of how the codes are related to each other.^50^, ^52^ Later, three of the authors (LLP, BB and HES) checked for consistency within codes applied and contrasting meanings across the full data set (Stage 5) ahead of confirming final themes and sub‐themes (Stage 6).

Despite English being the language of education in Singapore, Singaporeans commonly use fractured, ungrammatical English, locally known as Singlish.^54^ Singlish is a cultural marker for many and has evolved due to the influence of the diverse dialects spoken locally. For clarity, the narratives reported in this paper have been linked together with pronouns and verbs using square brackets so that the quotes transpose to coherent written text. For example: ‘I [would] go with anybody, helping; talking about this, that, everything… If there was someone to bring me out, chitchat with me, [it] would make me happy also*’*.

Detailed reflexive notes, in the form of contact summaries,^55^ were made as soon as possible post‐interview. The notes included observations about the quality of the interaction and any tensions, questions asked by the interviewee and unexpected themes that might warrant checking out in subsequent interviews.^56^ The process of reflexivity continued during the analysis in the form of memos.^57^, ^58^ Incorporated into this was the process of bracketing, that is identifying and setting aside researchers' assumptions so as to limit in so far possible prejudices and assumptions that might influence interpretation of the data.^57^, ^58^

Analysis was iterative and concurrent with data collection,^59^ repeatedly moving from the specific details of the data to abstract, theoretical constructions.^60^ Data saturation was based on inductive thematic saturation within topics.^61^

### 2.2 Public involvement in this study

At the time of this study, there were no frameworks for PPI in Singapore. This study was intended to engage with members of the public to explore their views as a first step in addressing that gap. It was itself an extended PPI consultation.

## 3 RESULTS

### 3.1 Participants

Twenty people participated (11 women; 9 men) with an average age of 47 years (range 26‐71). Six people were ≥ 60, of whom four had been involved in designing a study to develop models of intergenerational support. Eleven people were Singaporean Chinese, four were Singaporean Indian, and three were Singaporean Malay. Four were retired, 15 were working, and one was a housewife (Table 1).

**Table 1:** Participant characteristics

	Gender	Age	Ethnic group	Education level	Profession/employment status	Participated in medical research before	Previous PPI involvement
P01	F	67	Chinese	Secondary School^a^	Retired (accountant)	Yes	No
P02	M	34	Chinese	PhD	Research Fellow (Materials Science)	No	No
P03	M	32	Chinese	Masters	Research Associate (Physics)	Yes	No
P04	M	55	Chinese	Primary School	Taxi Driver	No	No
P05	F	53	Indian	Undergraduate	Teacher	No	No
P06	F	32	Chinese	Undergraduate	Public Relations Specialist	No	No
P07	F	66	Indian	Primary School	Housewife	No	No
P08	M	29	Indian	Secondary School	Journalist	No	No
P09	F	26	Malay	Undergraduate	Administration	No	No
P10	F	37	Caucasian^b^	Undergraduate	Teacher	No	No
P11	F	60	Chinese	Masters	Retired (Teacher)	Yes	Yes
P12	F	31	Chinese	Undergraduate	Marketing	No	No
P13	F	61	Chinese	Secondary School	Retired (Quality Control Inspector)	Yes	Yes
P14	M	71	Chinese	Secondary School	Retired (stock clerk)	Yes	Yes
P15	F	64	Chinese	Secondary School	Third Sector Worker	Yes	Yes
P16	M	59	Filipino^c^	Graduate (diploma)	Engineering Technician	No	No
P17	M	56	Chinese	PhD	Researcher (Marketing)	No	No
P18	M	37	Malay	Graduate (diploma)	Businessman	No	No
P19	F	30	Indian	Undergraduate	Social Worker	No	No
P20	M	37	Malay	Graduate (diploma)	Teacher	No	No

^a^ Singaporeans are 15‐16 years old when they complete secondary education.

^b^ Singaporean citizen at the time of the interview.

^c^ Singaporean permanent resident at the time of the interview.

The average interview lasted approximately one hour. The total amount of audio data was 19 hours. There was consistency in views between the perspectives of those who had previous experience of PPI (who shared their experiences) and those who had never been involved in research (who shared their expectations). The only difference between the two types of interviewees was that those with experience of involvement were able to give details of how that had operationalized in reality.

### 3.2 Themes

Four main themes emerged from these interviews:

- potential benefits
- challenges
- facilitators
- strategies for implementation.

Each of these themes is presented together with the sub‐themes that structure them using illustrative quotations. Unique identifiers include participant number, age, gender and ethnicity, with previous experience of involvement in health research indicated by yes or no (‘Y’/’N’).

#### 3.2.1 Potential benefits of PPI

All interviewees recognized the potential benefits of PPI. These were structured around three sub‐themes: positive impact on research, benefits for those involved and benefits for researchers. Whilst people identified a number of challenges around motivation to be involved, all described it as a worthwhile exercise.

##### Positive impact on research

The first sub‐theme was rooted in a realization of the need for including the perspectives of those whom the research is intended to help, that is patients and the public: ‘*It will move us forward, because after all research is meant for everyone, and not just researchers*’ (P17/56/M/Chinese/N).

Linked to this was its potential to strengthen research through community engagement, as this participant with previous experience reflected: ‘*You have to step into the community to do better research’* (P13/61/F/Chinese/Y). At a practical level, PPI was perceived as beneficial in all phases of research, from preparation of funding applications, through to data interpretation and implementation of evidence. Funding applications could be strengthened by ensuring that patient knowledge informed the questions and design: *‘If you have the inputs of the people on the ground, it will probably be beneficial to the funding application’* (P18/37/M/Malay/N). It was anticipated that PPI could enrich the interpretation of data and help reduce bias attributable to seeing things only from the perspective of researchers, referred to as the ‘*researcher's lens’* (P19/30/F/Indian/N). In terms of implementation, PPI was seen to have the potential to influence policy and service stakeholders who would be more responsive to recommendations from a study with public collaboration: *‘[The] general public, what they feel is very important for someone to implement something’* (P04/55/M/Chinese/N). In terms of meaningful public involvement, it was also considered that PPI had a role to play in ensuring the study design was such that participants felt valued and included, and thus would be more likely to engage fully: *‘I think people might give more instead of just being a passive participant’* (P20/37/M/Malay/N).

##### Benefits for those involved

Linked to this more active participation, interviewees not only saw the potential for PPI to improve research, but also expected it to have an impact on themselves and others taking part. Within this theme, some generational differences emerged. Younger interviewees considered it an opportunity to improve professional prospects and gain extra income:

Students, it's very clear cut. They [we] want credit. They want to be able to put it on their resume like; ‘I helped the researcher do that’. A bit of pocket money would definitely help. (P08/29/M/Indian/N)

The expectations on the part of older interviewees, however, centred more on self‐fulfilment and the hope that it would provide stimulation and opportunities to gain skills and knowledge, and increase self‐confidence: ‘*[…] stimulate [your] mind, make [you] work and think. […] give you the opportunity to improve yourself […] more confident*’ (P14/71/M/Chinese/Y). It could also provide experiential learning opportunities, developing new insights into the research process and the role of research in health: ‘*A different way of looking at researchers [and through that] research can be very interesting’* (P11/60/F/Chinese/Y). However, the importance of providing practical resources to support involvement was also recognized, as highlighted by one interviewee, who was physically disabled:

I [would] go with anybody, helping; talking about this, that, everything … If there was someone to bring me out, chitchat with me, [it] would make me happy also. (P07/66/F/Indian/N)

##### Benefits for researchers

Beyond the more practical considerations of design and recruitment, participants also saw benefits of PPI for researchers, in its potential to enhance understanding of the impact of research on patients:

It's easy to be caught up in your own circles, and it's not often that you see how what you do impacts the layperson. (P08/29/M/Indian/N)

There was also a recognition of the need for a change in attitudes amongst some researchers, with a sense that not all are prepared to involve the public in their work. This was seen to be linked to limited training and resources:

I don't know whether researchers have the luxury of the time to do it and even the skillset to do it [effective PPI]. (P06/32/F/Chinese/N)

However, they saw the development of such skills as having positive outcomes by enabling researchers to sensitively and appropriately work with diverse ethnic groups and with community members, to identify culturally specific issues and solutions:

[Researchers] do not know that these are the sorts of cultural issues […] involving people in the community helps […] being able to reach out to more people able to give you inputs that you do not have. (P20/37/M/Malay/N)

This was seen as particularly important in the context of Singapore, which has a substantial number of non‐local researchers, for whom ‘*it may help to have a Singaporean around to help’* (P09/26/F/Malay/N).

#### 3.2.2 Challenges to PPI

Despite the potential benefits of PPI identified, interviewees also mentioned potential challenges. However, no concerns were raised over potential individual harm or risk. The challenges consisted of three sub‐themes: pragmatic citizenship, the education system and deferential attitudes.

##### Pragmatic citizenship

Interviewees described their compatriots as predominantly pragmatic and passive in terms of social and community engagement: ‘*They don't like to talk about things, they are more Asian culture. They just live their life’* (P03/32/M/Chinese/N).

This had broad implications in terms of how people engage with issues around individual and collective morality and social norms:

Singaporeans in general are apathetic about the issues of ethics, principles and conversations that go beyond bread‐and‐butter issues. (P08/29/M/Indian/N)

Such pragmatism was rooted in practical issues that reflected attitudes to society and work which were felt to potentially deter people from PPI, particularly when there was no personal benefit: ‘*For me, I’m quite pragmatic, so I’ll do it on a monetary [basis], unless I’m one of the patients*’ (P03/32/M/Chinese/N), with a lack of time sometimes being used as an excuse: ‘*I’m not sure if they are genuinely busy or if it's just an excuse*’ (P19/30/F/Indian/N).

In terms of culture, it was considered that in Singapore, people are less open and transparent with each other than in other societies, with implications for meaningful involvement: ‘*Human to human interaction and relations are probably not as open as it would be in other countries*’ (P09/26/F/Malay/N).

This reserve was seen as a result of the need to avoid potential humiliation and thus an individual speaking out only when certain they are right. One participant expressed this in terms of a Chinese Hokkien term, ‘kiasu’, used colloquially in Singapore to refer to anxious, selfish behaviour characterized by a fear of ‘missing out’ or ‘losing out’.^62^ This term is closely linked with ‘kiasi’ which means taking extreme measures to avoid risk:

Social circles tend to be very tight. It's very hard to get a Singaporean to open up [it is] the kiasu culture, then everyone is scared to lose face. (P12/31/F/Chinese/N)

##### The education system

Underpinning such a culture is the education system which participants considered generates high levels of literacy whilst producing students who are required to show respect and not challenge their teachers. Consequently, critical thinking is often poorly developed: *‘Our school system kind of teaches people the mechanics of education but discourages people to think independently’* (P17/56/M/Chinese/N), resulting in what a number of participants described as the ‘*Asian mindset’* (P02/34/M/Chinese/N).

Linked to this, the education system was not considered to give students an understanding of research: *‘We have some kind of programme which allows the students to research, but […] it doesn't give them a real sense of what research is’* (P20/37/M/Malay/N).

It was considered that this contributed to research being seen as the domain of researchers: *‘The individual researcher's piece of work. It doesn't involve public. It's very personal. There's nothing public about it’* (P11/60/F/Chinese/Y), with the role of the public confined to passive participation and a means to an end: ‘*[…] ‘the white mouse’. For what? They do a test. It's like guinea pig*’ (P14/71/M/Chinese/Y).

##### Deferential attitudes

Linked to this notion of research being the domain of researchers only, the culture of deference embedded within the education system was seen to lead to assumptions and beliefs around the status of researchers that positioned them high in the social order:

A lot of Singaporeans think research is for people who are way up there. They have to have a very high pay; they have to be super smart and stuff like that. (P09/26/F/Malay/N)

The implications of such hierarchical positioning were intensified by the character trait of ‘saving face’ discussed above and were seen as a barrier to laypeople's genuine engagement with the research process:

Sometimes they [participants] see researchers as, ‘oh, he is so educated, right? How can I share with him because I feel very small?’ […] they might not want to share with them because they don't want to lose face. (P10/37/F/Caucasian/N)

Such deference could result in an unquestioning position, which would limit the impact of involvement:

Because their qualification is very [high]… They study about it. So, we don't know about it, which is wrong, and which is right. What he [researcher] says, we follow. (P07/66/F/Indian/N)

#### 3.2.3 Facilitators for PPI in Singapore

Despite these challenges, interviewees also highlighted characteristics of Singapore society that could facilitate involvement locally. These consisted of two sub‐themes: the ageing population and cultural change.

##### The ageing population

Firstly, the rapidly ageing population was perceived as a potential catalyst for PPI adoption, particularly as the associated health‐care demands necessitate research focusing on innovations and interventions tailored to the needs of older people. The life‐experiences of older people were seen to provide an added dimension which could enrich research: ‘*Youngster just come out, so they know less than what the old people know’* (P07/66/F/Indian/N).

Interviewees considered the wish to help others as a strong motivation for older people. This was thought to be rooted in the ‘kampung spirit’,^63^ generally considered to apply amongst older generations and used in Singapore to describe a sense of social cohesion that is rooted in the Malay notion of traditional village life: *‘[PPI] is a way I can use to help myself. I find it useful, so I can use it to help others’* (P11/60/F/Chinese/Y).

##### Cultural change

Secondly, despite the cultural challenges, interviewees recognized Singaporean society is changing in ways that will create more opportunities for PPI, with people becoming less closed and wanting to contribute more at a community and societal level. They described increasing interactions amongst people from differing ethnic communities, and across age groups, and saw ways in which PPI could both be nurtured and further nurture these new relationships. This move to diversity and inclusion was observed amongst individuals and government:

[Authorities] are making moves towards doing that [giving people a voice], but they should be more radical in it. (P08/29/M/Indian/N)

Although hierarchical attitudes reinforced by the education system were seen as challenges, on the other hand the increasingly high level of education could mean people were better able to contribute to research. Furthermore, the emphasis on discipline and respect for authority was considered consistent with the systematic requirements of best research practice and thus facilitators for involvement:

Because Singaporeans are very structured. People could [do] research a little bit better because they like to follow rules. (P10/37/F/Caucasian/N)

#### 3.2.4 Strategies for implementation

During the interviews, participants drew on their past experiences of campaigns driving changes in health care to suggest both formal and informal strategies to realize PPI in Singapore. A frequently cited example was the recent government‐led initiative to address diabetes by involving communities and voluntary organizations^64^:

##### Formal strategies

Interviewees discussed the potential to introduce policies to promote PPI which, given the culture of deference, most interviewees considered could be achieved by making it mandatory: *‘That would make a whole world of difference and should be implemented right away’* (P17/56/M/Chinese/N). Such potentially ready engagement by the public was seen as a benefit of a style of government that emphasizes the collective rather than the individual: *‘Just one of the perks of being semi‐authoritarian in Singapore, because it works’* (P08/29/M/Indian/N).

However, linked to this there was some concern that a top‐down approach would defeat the principle of empowerment inherent within PPI, particularly undermining the potential to identify research priorities, resulting in research questions that did not challenge existing policies and practices:

Sure, there are certain things that are purely medical that do not have huge political or social implication. Those are usually allowed, and the moment they have some level of social political impact, then they are very controlled. (P17/56/M/Chinese/N)

##### Informal strategies

Secondly, interviewees suggested various informal strategies to support PPI implementation, beginning with raising awareness. The role of the community was highlighted, with those with PPI experience recalling researchers effectively engaging and collaborating with community members, building trusting relationships to foster confidence amongst those involved:

[Study name], they are very good. They know the need, between the young people and the old people, and they use a very attractive way, a creative way of bringing us together. The young girl that partnered me, we are still in touch. She called me. I went out to lunch with her. At first, I talked one hour, [then] we talked for four hours. […] So, for me […] you're creative in helping the elderly to feel more confident to want to participate. (P15/64/F/Chinese/Y)

Personal contact with researchers in familiar environments, with opportunities to informally ask questions, was considered important ahead of making a decision as to whether or not to engage: ‘*People who are interested to learn [could] sit down with researchers and whatever, and then they can volunteer*’ (P06/32/F/Chinese/N).

As part of this rapport building, the importance of using lay terminology and avoiding highly technical explanations was highlighted, including being clear about the research aims, and the role and purpose of PPI in particular projects:

It sounds like a very scary thing. Rocket science. But, if you can break down to them, really, what research really is about, and how they can involve themselves…. (P19/30/F/Indian/N)

Interviewees identified the importance of emphasizing PPI as a shared collaboration between people, research communities and the government:

You have to start from the bottom up […] we are still Asian […] still quite collective at a certain level, even though we try to be individualistic. (P17/56/M/Chinese/N)

Indeed, most interviewees thought that promoting PPI ‘*should be a joint effort between the community, the research institutes and the government’* (P08/29/M/Indian/N).

##### Learning from community organizations

Finally, it was suggested that researchers could learn from community organizations that are already using bottom‐up strategies and inclusion to develop their understanding of community participation:

If you are talking about lay people, then you should try everybody. You can start with [community] organisations. (P17/56/M/Chinese/N)

Such community groups were seen as an obvious means of identifying research advocates:

You need to find the leaders. You need to go to communities that you see that are very active in their monthly gatherings. Those are the people that might be willing to help you do this. (P10/37/F/Caucasian/N)

A community‐focused approach was also seen to be reflected in recent shifts to emphasize a shared identity, encouraged by government policy and supported by community initiatives:

This desire of getting to know each other […] Last time [before now] we just kept quiet, but now we interact here and there. So, there is this effort of wanting to know, to continue to maintain this harmony that we have. (P15/64/F/Chinese/Y)

Finally, it was considered important to develop a range of communication strategies to engage different populations and lifestyles. Such strategies should include social media and mobile applications:

If an app could be created, social media is being used, like a cool, attractive one, or something, then you'd probably get the younger generation. (P19/30/F/Indian/N)

## 4 DISCUSSION

### 4.1 Summary of findings

Findings from this study suggest that although laypeople in Singapore may be unfamiliar with the concept of PPI, once it was explained they recognized its potential benefits at both an individual and a societal level. PPI was thought to offer the potential to generate individual and collective empowerment and to build new relationships and trust between laypeople, academics and health professionals. Proposed challenges were seen as culturally rooted in the ‘*Asian mindset’*, because of social norms emphasizing a high degree of deference, an educational system resulting in high literacy and numeracy but low critical appraisal skills, and a society with a heavy emphasis on long‐working hours to the detriment of community involvement. Nonetheless, interviewees noted current cultural shifts which could facilitate PPI adoption, including the revival of a ‘*kampung spirit’* with its emphasis on community support and inclusion. Participants identified formal and informal implementation strategies, including government policies and incentives and awareness‐raising campaigns at a community level.

However, PPI has been criticized for being dominated by top‐down and expert‐driven models^65^ that do not address the power imbalance between professional and lay experts.^66^, ^67^, ^68^, ^69^ These concerns were reflected in the views of participants in this study, with concern that not all of the many approaches would yield participant empowerment or improving research.^70^, ^71^, ^72^, ^73^ As a consequence, such approaches continue to fall short of meeting the ethical drivers implicit in PPI.^2^, ^15^, ^74^, ^75^, ^76^

### 4.2 Possible way forward: A supported group model of involvement

**Figure 2:**
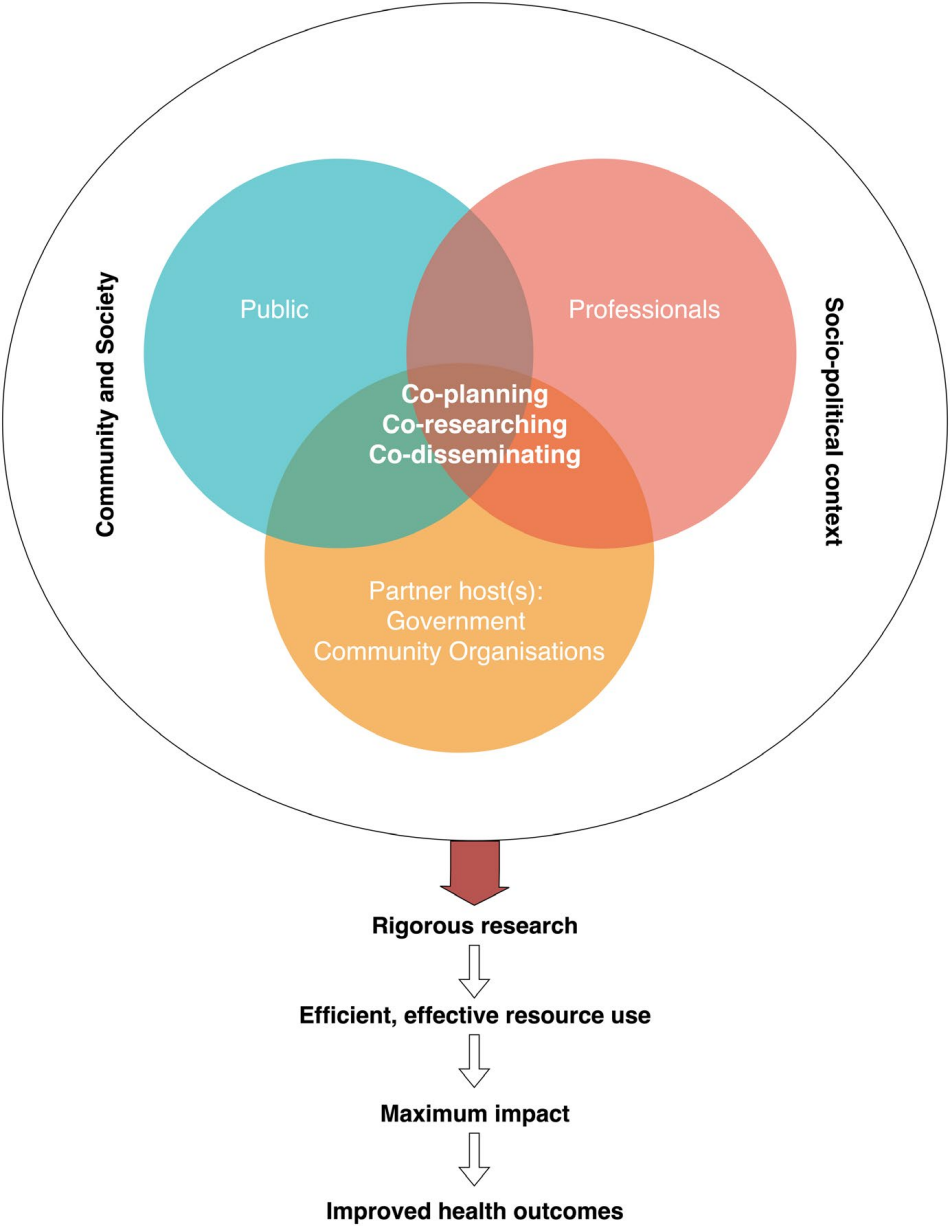
Supported group model for PPI in health research based on Ref. 76

Previous studies suggest that it is the structure of engagement that determines who gets involved, and to what outcome.^76^, ^77^ From this, it would seem that a structural approach reflecting Singaporean society and culture may help in implementing PPI and maximizing its potential locally, for which an adapted version of the ‘supported group model’^77^ may offer a useful framework. Following this model, a partnership between researchers, the Singapore Government and community organizations would allow for progressive and sustained involvement of the public in research. This model is aligned with the government's policy of ‘Shared Values’^35^, ^37^ intended to foster community cohesion, as well as reflecting the structure of Singaporean society.^29^, ^78^ Whilst the latter acknowledges the importance of expertise, the former emphasizes the importance of the state (‘*Nation before community and society above self’*) and communities (‘*Community support and respect for the individual’*).^37^ The model reflects three components or key players: researchers who hold technical knowledge and expertise, communities with lay knowledge and expertise, and the government and other civil organizations which form a mediator‐partner host (Figure 2).

Underpinning such a model is the need to educate researchers on the role and importance of PPI,^41^ and upskill them in strategies to engage and involve at community and individual level through collaboration and consultation.^79^ Inherent within this is the need to develop strategies to address issues of power imbalance, exacerbated in the hierarchical structure of Singaporean society,^29^, ^80^ where the public rarely challenges the perceived expertise and authority of professionals or researchers. As participants also emphasized, strategies for engagement and involvement need to be rooted in local culture yet remain responsive to what is a rapidly changing context. Linking to this, findings highlight the need to support a shift in the understandings of patients, the public, professionals and policymakers on the importance of collaboration and partnership in prioritizing the research agenda, designing relevant research and maximizing impact to improve health at an individual and a community level.

### 4.3 Strengths and limitations of this study

This is the first study to explore the perspectives of the Singaporean public on PPI, drawing on a range of views, from diverse ages and ethnic backgrounds. It identifies promising opportunities for PPI in an Asian setting, whilst also highlighting a number of challenges that are culturally specific. The findings from this study, including the supported group model, can be extrapolated internationally to multicultural societies where engaging ethnically diverse groups in research remains an ongoing challenge.^25^, ^27^, ^81^, ^82^, ^83^

A further strength of the study was the contribution of researcher reflexivity. The use of the contact summary forms, memo writing and discussions with the other authors encouraged further reflection. This process informed both data collection (refining the topic guide) and analysis. The study was undertaken as part of a doctoral research project by LLP, a non‐Singaporean. Initially, this led to concern that the public would be reluctant to share their views with someone perceived as an outsider. In fact, participants shared generously, often commenting that they felt less constrained talking to an outsider who might be better positioned to critically evaluate the situation in a way that a Singaporean might not. Consequently, being an outsider generated potentially richer data than might otherwise have been the case.

In terms of limitations, this was a small exploratory study which showed no differences in attitudes across the ethnic groups included. More in‐depth exploration of attitudes across a diverse range of ethnic populations is needed in order to fully understand the potential for PPI in Asia.

## 5 CONCLUSION

Our study concludes that whilst unaware of the concept of PPI, participants recognized its potential to increase impact and improve implementation of research findings into health care, as well as having direct personal benefits for engaged individuals. Interviewees noted challenges specific to the local context, particularly hierarchical power relationships between professionals and the public. Facilitators included current demographics, cultural shifts and a tendency to comply with centralized initiatives, leaving room for both formal and informal PPI implementation strategies.

Findings suggest the need for a ‘supported group model’ of involvement. Such a model offers opportunities for partnership and collaboration framed within the socio‐political context of Singapore, within which the principles of PPI resonate deeply with current community‐based initiatives from diverse stakeholders, including the government. However, given the lack of familiarity amongst the public of research generally, and PPI specifically, a combination of formal and informal strategies will be required to raise awareness of the unique contribution such involvement makes to research. More research is needed to understand the motivators and barriers to involvement amongst diverse ethnic populations in Singapore and in Asia, and to identify culturally sensitive solutions. Finally, careful consideration needs to be given to issues of resource and support, both educationally and practically, so as to enable members of the public to engage with researchers in meaningful ways that will deliver research best placed to improve patient outcomes.

## CONFLICT OF INTEREST

There was no financial support or other benefits from commercial sources for the work reported on in the manuscript, or any other financial interests that any of the authors may have, which could create a potential conflict of interest or the appearance of a conflict of interest with regard to the work.

## ETHICS

The study was reviewed and approved by the University's Internal Review Board, April 2018.

## Data Availability

The data that support the findings of this study are available from the corresponding author upon reasonable request.
